# Predicting progression of Alzheimer's disease

**DOI:** 10.1186/alzrt25

**Published:** 2010-02-23

**Authors:** Rachelle S Doody, Valory Pavlik, Paul Massman, Susan Rountree, Eveleen Darby, Wenyaw Chan

**Affiliations:** 1 Alzheimer's Disease and Memory Disorders Center, Baylor College of Medicine, 6501 Fannin Street, NB302, Houston, TX 77030, USA; 2 Division of Family Medicine, Baylor College of Medicine, 3701 Kirby Drive, Houston, TX 77098, USA; 3 Department of Psychology, University of Houston, 126 Heyne Building, Houston, TX 77204-5022, USA; 4 Department of Epidemiology and Biostatistics, University of Texas Health Sciences Center, 7703 Floyd Curl Drive, San Antonio, TX 78229-3900, USA

## Abstract

**Introduction:**

Clinicians need to predict prognosis of Alzheimer's disease (AD), and researchers need models of progression to develop biomarkers and clinical trials designs. We tested a calculated initial progression rate to see whether it predicted performance on cognition, function and behavior over time, and to see whether it predicted survival.

**Methods:**

We used standardized approaches to assess baseline characteristics and to estimate disease duration, and calculated the initial (pre-progression) rate in 597 AD patients followed for up to 15 years. We designated slow, intermediate and rapidly progressing groups. Using mixed effects regression analysis, we examined the predictive value of a pre-progression group for longitudinal performance on standardized measures. We used Cox survival analysis to compare survival time by progression group.

**Results:**

Patients in the slow and intermediate groups maintained better performance on the cognitive (ADAScog and VSAT), global (CDR-SB) and complex activities of daily living measures (IADL) (*P *values < 0.001 slow versus fast; *P *values < 0.003 to 0.03 intermediate versus fast). Interaction terms indicated that slopes of ADAScog and PSMS change for the slow group were smaller than for the fast group, and that rates of change on the ADAScog were also slower for the intermediate group, but that CDR-SB rates increased in this group relative to the fast group. Slow progressors survived longer than fast progressors (*P *= 0.024).

**Conclusions:**

A simple, calculated progression rate at the initial visit gives reliable information regarding performance over time on cognition, global performance and activities of daily living. The slowest progression group also survives longer. This baseline measure should be considered in the design of long duration Alzheimer's disease clinical trials.

## Introduction

There is considerable variability in progression rates among Alzheimer's disease (AD) patients. Patients and families frequently ask clinicians to prognosticate regarding expected rates of cognitive and functional decline, and clinicians have little basis for making such predictions. We have shown that it is possible to reliably estimate early AD symptom onset, and together with baseline MMSE score, to calculate a rate of progression at the initial assessment (the pre-progression rate) [1,2]. The use of a rate to estimate early progression gives information on severity, but also on how long it took for the patient to reach the current severity level, which reflects that individual's disease characteristics better than a severity score alone. However, it is not clear whether patients maintain a similar rate of decline throughout the course of their disease or change trajectories over time, due to endogenous or exogenous factors (such as treatment). Demonstrating the predictive value of the calculated pre-progression rate would be valuable for patient and family counseling, as well as for providing a research marker of phenotypic variability to validate biological markers of progression. Further, the ability to model group progression of AD patients is essential for designing disease-modification studies of new AD treatments, and pre-progression might be an important baseline variable to take into account in the analysis of clinical trial data [3].

The Baylor Alzheimer's Disease and Memory Disorders Center has followed a cohort of AD patients for up to 15 years, with detailed clinical and neuropsychological data obtained at baseline and at annual follow up visits which are maintained in an ongoing electronic data base. We used these data to answer the following questions: 1) does a pre-progression rate calculated at the initial assessment predict subsequent performance in specific cognitive and functional domains during follow up, and 2) is the pre-progression rate associated with overall survival, after adjustment for relevant covariates?

## Materials and methods

The Baylor Alzheimer's Disease and Memory Disorders Center sees self-referred, agency-referred, and physician-referred individuals for evaluation and management of cognitive complaints. We evaluate patients for systemic and brain disorders with laboratory testing, including neuroimaging, and psychometric tests. We assign a diagnosis of various subtypes of mild cognitive impairment (MCI) or dementia according to standardized criteria through a consensus conference [4,5]. Details of the Baylor ADMDC patient recruitment, assessment, follow up procedures, and long-term clinical outcomes in the patient cohort have been reported [5]. Patients who meet standardized diagnostic criteria for probable or possible Dementia with Lewy Bodies are excluded from the Probable AD diagnostic category. Patients included in this analysis are enrolled in the Baylor Alzheimer's Disease Center and the database has been approved by the Baylor Institutional Review Board. Patients and/or their legally designated representative sign consent for storage and use of their data.

### Measures

Cognitive outcome measures routinely obtained at baseline and at annual follow up include the Mini Mental Status Exam (MMSE), [6] a widely used dementia severity test with scores ranging from 0 to 30 points, and the Alzheimer's disease Assessment Scale-Cognitive Subscale (ADAS), [7] a measure of cognitive domains often impaired in AD including memory, orientation, visuospatial ability, language, and praxis. Scores range from 0 to 70 with higher scores reflecting more cognitive impairment. Attention and concentration are assessed with the Verbal Series Attention Test (VSAT) [8]. This test consists of forward and reverse generation of arithmetic series, verbal series (for example, months of the year), number-letter sequencing and auditory vigilance for a spoken target letter and is scored for time taken to complete each task (up to 480 seconds) and the number of errors made (up to 45). To assess global performance we use the Clinical Dementia Rating Scale Sum of Boxes (CDR-SB) [9,10]. This score is derived from a patient interview and mental status examination in conjunction with an interview of a collateral source. The CDR-SB score (range 0 to 18) is obtained by summing ratings in each of six cognitive domains or *boxes *including memory, orientation, judgment/problem solving, community affairs, home and hobbies, and personal care. Higher scores reflect more global impairment. Functional outcomes are assessed with the Physical Self-Maintenance Scale (PSMS) and Instrumental Activities of Daily Living scale (IADL), which together constitute the Lawton and Brody Activities of Daily Living Scale [11]. The PSMS quantifies difficulties with basic activities of daily living such as eating and dressing, and each item is scored from 1 to 5 with a maximal score of 30, representing maximal impairment. The IADL evaluates eight complex daily living tasks such as the use of the telephone, ability to shop, and to make use of transportation. Scores range from zero to 31, with higher scores indicating more functional impairment.

Covariates previously reported to influence progression in AD and routinely collected at the baseline visit are pre-morbid IQ estimated by the American version of the New Adult Reading Test (AMNART) [12,13], age, sex, years of education, history or presence of hallucinations, delusions, and extra-pyramidal signs [14,15]. In our previous work, premorbid IQ was a better predictor of progression rates than education [16], and this was taken into account in the modeling described below. We used a modification of the motor scale of the Unified Parkinson's disease Rating Scale to capture extra-pyramidal signs [17].

Vital status is obtained from the National Death Index every six months, with a censoring date on December 31, 2004.

### Calculation of pre-progression rate

The pre-progression rate is calculated using a clinician's standardized assessment of symptom duration in years and the baseline MMSE. We obtain the clinician estimate of duration using a standard procedure which includes a series of questions about the duration of specific symptoms that might be a sign of AD, combined with medical records review, an informant interview, and hypothesis-testing. Inter-rater reliability for the estimate is 0.95 [2]. Since a cognitively intact individual should obtain the maximum MMSE score of 30, the pre-progression rate is given by the formula: (30 - baseline MMSE)/estimated duration of symptoms in years. Patients with an MMSE decline of less than two points per year are classified as slow progressors, between a two- to four-point decline as intermediate progressors, and more than or equal to five points per year as rapid progressors [1]. In a previous study, we found that use of a normed MMSE score, based upon age, education, and gender [18] underestimated the baseline MMSE score for 7% of the subjects [1], which is why we have adopted the maximal score of 30 in our formula. Since MMSE decline is non-linear, we used groupings of MMSE change rates (slow, intermediate, rapid) which are more clinically relevant than absolute rates of change (for example, one point per year is really not clinically different from two points per year because of test-retest variability).

### Patient inclusion criteria

Only probable AD patients (NINCDS-ADRDA, DSM IV) were included. Patients had to have a pre-progression index calculated at baseline, an AMNART score, and at least one comprehensive follow-up visit approximately one year later.

The first patient was enrolled in 1989, and accrual has been ongoing since then. The AMNART was incorporated in 1994. The ADAS-Cog, PSMS, and IADL scales were not used routinely until 1995, whereas other outcome measures were collected in earlier years. Rather than requiring all patients to have all of the outcome measures, we allowed individuals to enter each analysis if they had a measure of the outcome in question and non-missing values on the adjustment covariates. We report in the Results section the number of persons included in each regression equation.

### Statistical analysis

The study data are longitudinal, with fixed values associated with demographic characteristics and baseline clinical presentation, and time varying values on cognitive and functional outcomes. For the analysis of progression of AD, we used random effects linear regression models to estimate the relationship between the pre-progression categories and the rate of change in the ADAS-Cog, VSAT Time, VSAT Errors, CDR Sum of Boxes, PSMS and IADL scores [19]. Coefficients yielded by this type of model reflect the change, or slope, in the outcome for each unit change in a predictor variable, holding values of the other variables in the model constant. The random effect is time in years, and we used a time by pre-progression rate interaction term to indicate whether or not there is a difference in average rate of decline (slope) associated with a patient's initially calculated pre-progression group. A significant time by pre-progression rate interaction term could represent divergence among the groups in rates of change. We examined each model for significance of a quadratic term and used non-linear interactions when the quadratic was significant (but report both the linear and non-linear interactions in Table 1). Potential confounders or effect modifiers of the association between cognitive or functional outcomes and the pre-progression rate included age, sex, race/ethnicity (non-Hispanic whites vs. Hispanic whites, blacks and other ethnicities), years of education, AMNART score (as a measure of pre-morbid IQ), and baseline clinical features of history or presence of hallucinations, delusions, and Parkinsonian signs. Each covariate was evaluated in a base model that included baseline severity (dichotomized as mild or moderate-to-severe based on MMSE score), duration of symptoms, and pre-progression rate categories (slow, intermediate, fast). For the baseline covariate, the moderate and severe groups were combined (MMSE <20) since there were relatively few patients classified as severe at baseline. Covariates significant at the *P *< 0.10 level were included in a final model for each cognitive or functional outcome. Our analysis included data for up to seven years of follow-up, since this interval represented the 90^th ^percentile.

**Table 1:** Relationship between pre-progression category and subsequent rate of decline on cognitive and functional measures

	Progression measures											
Independent Variables¶	ADAS-Cog (n = 552)		VSAT Time (n = 589)		VSAT Errors (n = 589)		CDR-SB (n = 596)		IADL (n = 573)		PSMS (n = 575)	
	Beta	*P*	Beta	*P*	Beta	*P*	Beta	*P*	Beta	*P*	Beta	*P*
Duration of Symptoms	1.352	<.001	7.405	<.001	-0.778	<.001	0.446	<.001	0.523	<.001	0.243	.015
Baseline Severity (mild vs. moderate/severe)	-10.052	<001	-61.158	<001	-7.886	<.001	-3.088	<.001	-3.204	<.001	-2.129	<.001
Years of Follow-up	3.323	<.001	20.335	<.001	3.033	<.001	2.084	<.001	3.309	<.001	2.430	<.001
Years of Follow-up Squared	0.514	.036	--	NS	--	NS	--	NS	-0.207	.003	--	NS
Pre-progression Rate												
Intermediate vs. Fast	-4.032	.006	-20.351	.033	-3.046	.007	-1.399	.003	-1.915	.012	-0.442	.424
Slow vs. Fast	-9.458	<.001	-49.417	<.001	-6.533	<.001	-2.593	<.001	-3.051	.001	-0.454	.520
Linear Interaction 1*	--	NS	--	NS	--	NS	0.247	.039	--	NS	--	NS
Linear Interaction 2*	--	NS	--	NS	--	NS	--	NS	--	NS	-1.133	<.001
Non-linear Interaction1*	-0.807	.004	--	NS	--	NS	--	NS	--	NS	--	NS
Non-linear Interaction 2*	-0.554	.039	--	NS	--	NS	--	NS	--	NS	--	NS
												
Model Intercept	56.601		617.164		62.203		10.364		14.96		4.243	

* Interaction 1 = time by intermediate pre-progression group (fast = reference group); Interaction 2 = time by slow pre-progression group (fast = reference); Non-linear Interaction 1 = time squared by intermediate pre-progression group (fast = reference group); Non-linear interaction 2 = Time squared by slow pre-progression group (fast = reference group).

*¶ *Models adjusted for age at diagnosis, sex, years of education, duration of symptoms at diagnosis, baseline severity (categorical), pre-morbid IQ, and presence of hallucinations and/or delusions. If the quadratic term for follow-up time and the pre-progression group by quadratic time variable were not significant, coefficients for models with linear terms only are shown. Non-significant (NS) betas for interaction terms omitted from table.

ADAS-cog = Alzheimer's disease Assessment Scale-cognitive subscale; CDR-SB = Clinical Dementia Rating Scale Sum of Boxes; IADL = Instrumental Activities of Daily Living scale; PSMS = Physical Self-Maintenance Scale; VSAT = Verbal Series Attention Test.

Cox survival analysis with robust variance estimators for correlated observations was used to examine the contribution of baseline demographic variables, clinician's standardized estimate of duration, baseline AMNART score, and baseline MMSE score to annual risk of death. In the survival analysis, we considered the effect of each study variable alone and then in a full multivariable model. Using a conservative estimate, our study had 80% power to detect a reduction in hazard ratio of 32% (based upon N = 124 per group, medians of 8 and 10 years, type 1 error = 5% and Bonferroni correction).

All analyses were performed using STATA version 9.0.

## Results

Of 798 probable AD patients who met inclusion criteria, 597 had the AMNART as part of their initial baseline assessment. Since the AMNART was a pre-specified covariate, these 597 individuals formed the inclusion sample. Table 2 reports demographic characteristics and baseline test scores by preprogression group. From 34 to 46% of patients had a history of or current delusions at their initial visit, and 13 to 22% had a history of or current hallucinations, but only 3 to 7% had Parkinsonian signs on examination. It is notable that slow progressors had a longer estimated duration of symptoms than intermediate or fast progressors, consistent with slow progression. IQ and education were also higher in slow progressors. The distribution of APO E epsilon 4 alleles did not differ. Significant differences between the groups were taken into account in the analysis.

**Table 2:** Selected patient characteristics at baseline by preprogression category (n = 597)

Variable	Mean ± SD or n (Percent)			
	Fast (N = 124)	Intermediate (n = 274)	Slow (n = 199)	P *
Age at Diagnosis (years)	74.0 ± 8.7	73.6 ± 8.8	72.9 ± 8.2	.516
Sex (% female)	72.6	68.3	58.3	.016
Race/Ethnic Group (% white)	90.3	91.2	90.9	.957
Years of Education	13.0 ± 3.1	13.7 ± 3.1	14.4 ± 3.4	<.001
Estimated duration of disease before diagnosis (yrs)	1.7 ± 0.9	3.4 ± 1.6	4.9 ± 2.6	<.001
Baseline MMSE	18.1 ± 5.0	20.3 ± 4.4	24.7 ± 3.8	<.001
First AMNART (estimated IQ)	105.5 ± 9.8	106.3 ± 10.2	110.7 ± 9.6	<.001
Baseline MMSE	18.1 ± 5.0	20.3 ± 4.4	24.7 ± 3.8	<.001
Hallucinations (% yes at or before Baseline)	21.0	21.9	12.6	.027
Delusions (% yes at or before Baseline)	40.32	46.0	34.2	.035
Parkinsonian Symptoms at Baseline	6.5	4.4	3.0	.147
Number of APOE ε4 Alleles (% in each group)				
0	22.2	47.3	30.6	.573
1	19.4	46.2	34.4	
2	20.0	40.0	40.0	
ADAS Cog	27.4 ± 12.0	24.9 ± 11.0	17.6 ± 8.4	<.001
CDR Sum of Boxes	6.7 ± 3.9	6.0 ± 3.6	4.0 ± 2.8	<.001
PSMS	7.7 ± 2.5	7.7 ± 2.7	7.2 ± 2.2	.177
IADL	16.0 ± 6.8	15.2 ± 6.3	13.3 ± 5.5	.002
VSAT (time)	250.2 ± 91.6	229.15 ± 87.6	184.6 ± 73.7	<.001
VSAT (errors)	18.3 ± 11.8	15.0 ± 9.9	9.5 ± 8.1	<.001

*P-values based on one-way analysis of variance for continuous variables or Chi square test for categorical variables

ADAS-cog = Alzheimer's disease Assessment Scale-cognitive subscale; AMNART = American version of the New Adult Reading Test; CDR = Clinical Dementia Rating Scale; IADL = Instrumental Activities of Daily Living scale; MMSE = Mini Mental Status Exam; PSMS = Physical Self-Maintenance Scale; VSAT = Verbal Series Attention Test

**Figure 1:**
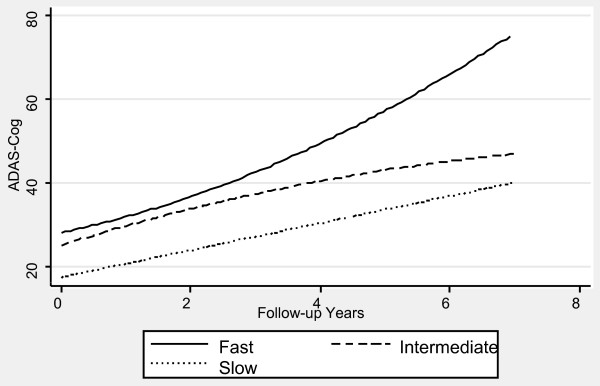
**Fitted regression lines for ADAScog by pre-progression category calculated from model coefficients shown in Table 1**.

**Figure 2:**
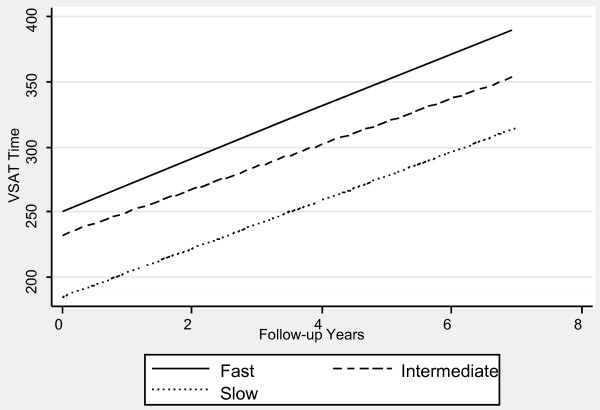
**Fitted regression lines for VSAT time by pre-progression category calculated from model coefficients shown in Table 1**.

**Figure 3:**
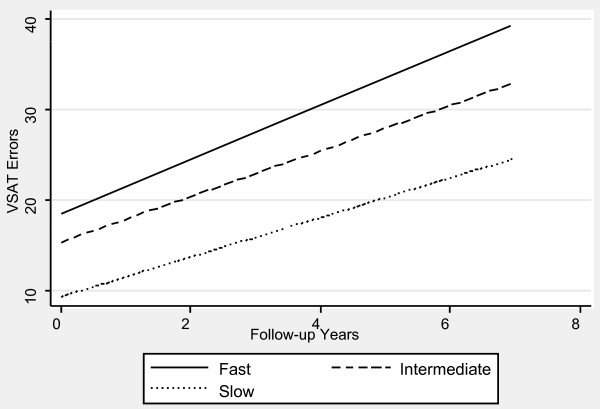
**Fitted regression lines for VSAT errors by pre-progression category calculated from model coefficients shown in Table 1**.

**Figure 4:**
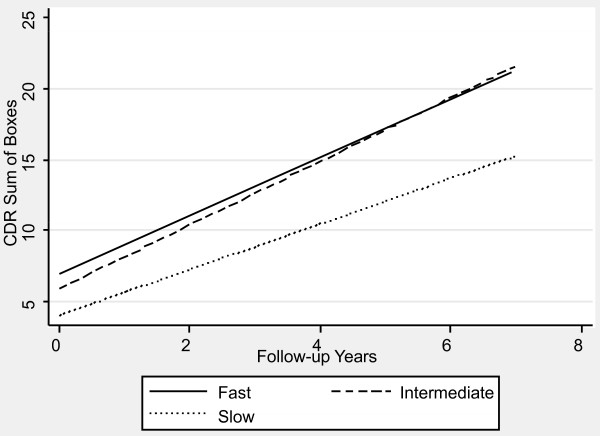
**Fitted regression lines for CDR-SB by pre-progression category calculated from model coefficients shown in Table 1**.

**Figure 5:**
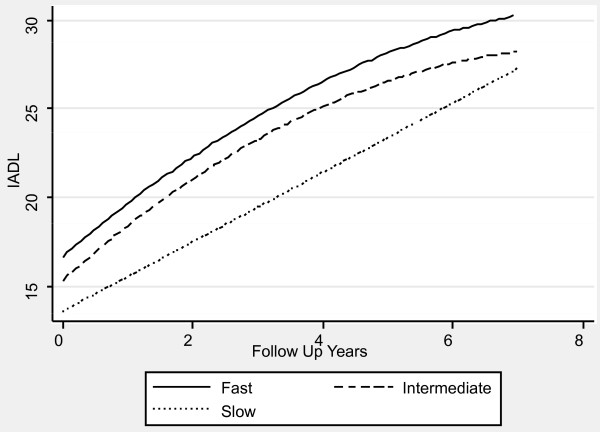
**Fitted regression lines for IADL by pre-progression category calculated from model coefficients shown in Table 1**.

**Figure 6:**
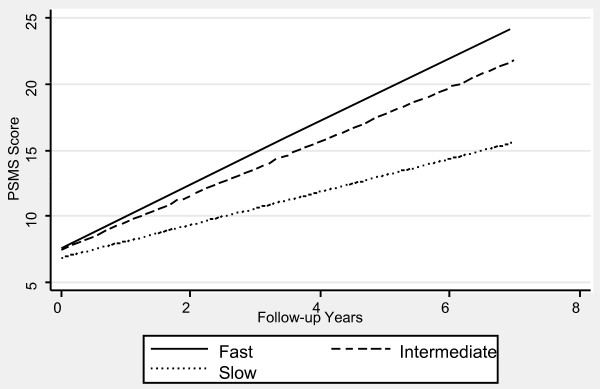
**Fitted regression lines for PSMS by pre-progression category calculated from model coefficients shown in Table 1**.

Table 1 contains the mixed effects linear regression coefficients associated with pre-progression categories and the interaction of pre-progression categories with time, after adjustment for the prospectively defined covariates. Figures 1, 2, 3, 4, 5 and 6 display the fitted regression lines predicted by the regression model for each outcome. Patients in both the slow and intermediated pre-progression groups maintained better performance on the ADAS-Cog, the CDR-SB, VSAT Time and Errors and the IADL, compared to fast pre-progressors, but showed no significant baseline difference on the PSMS. For example, slow progressors were about 9.5 points better and intermediate progressors four points better than fast progressors on the ADAS-Cog at baseline (Table 1). Over time, slow progressors gained 0.6 fewer points per year, and intermediate progressors gained 0.8 fewer points per year. Figure 1 shows that both of these groups diverged from the fast group over time. Similarly, slow progressors were 2.6 points lower and intermediate progressors 1.4 points lower on the CDR-SB to start with (Table 1). This relative difference between the slow and fast progressors was maintained (no significant interaction term), while the intermediate progressors gained 0.2 points per year more than the fast progressors, so that they caught up over time (Figure 4). This tendency of the intermediate group to speed up on the CDR-SB was probably not accounted for by functional deficits, since this did not occur on the IADL measure (Table 1 and Figure 5). Basic activities of daily living assessed by PSMS were not different at baseline and did not begin to diverge until the first couple of years of follow up (Table 1 and Figure 6), but the slower rate of worsening of the slow group (1.1 points less per year) led to more divergence from the fast group over time. Table 3 presents information on the relationship of the pre-specified covariates to each outcome. Not unexpectedly, age was related to cognitive scores, and sex to performance of complex ADLs. Pre-morbid IQ (AMNART score) was related to the cognitive measures. Education did not remain a significant predictor of progression on any measure in the presence of the AMNART, consistent with our previous findings [16]. The presence of delusions at or before baseline was associated with worse performance on all measures except the VSAT, and hallucinations at or before baseline were related to lower scores on measures that included activities of daily living. We did not find a relationship between any of our outcomes over time and the presence of baseline extrapyramidal signs in this population of probable AD subjects, from whom Dementia with Lewy Bodies was carefully excluded, and APO E genotype was not associated with the outcomes.

**Table 3:** Effect of covariates: betas (*P*-values) for significant covariates*

	Covariates							
Progression Measures	Age	Sex (1 = male, 0 = female)	Education	AMNART	Delusions	Hallucinations	Extra-pyramidal Signs	APOE Genotype
ADAS-Cog	-0.962 (.067)	NS	0.291 (.055)	-0.229 (<.001)	2.914 (.001)	NS	NS	NS
VSAT Time	-1.493 (<.001)	NS	NS	-2.339 (.001)	NS	NS	NS	NS
VSAT Errors	-0.179 (<.001)	NS	NS	-0.272 (<.001)	NS	NS	NS	NS
SCDR	NS	NS	NS	NS	1.386 (<.001)	1.245 (.003)	NS	NS
IADL	NS	-2.109 (<.001)	NS	NS	2.762 (<.001)	1.619 (.008)	NS	NS
PSMS	0.037 (.055)	NS	NS	NS	1.509 (<.001)	1.945 (.009)	NS	NS

*Betas calculated in models adjusted for baseline severity, duration, pre-progression rate × time, pre-progression × time squared (if applicable), and other covariates that achieved the selection criterion of *P *< 0.10. NS means the covariate did not achieve the criterion of *P *< .10, or did not retain this significance level when included in the full model.

ADAS-Cog = Alzheimer's disease Assessment Scale-Cognitive Subscale; AMNART = American version of the New Adult Reading Test; CDR-SB = Clinical Dementia Rating Scale Sum of Boxes; IADL = Instrumental Activities of Daily Living scale; PSMS = Physical Self-Maintenance Scale;  VSAT = Verbal Series Attention Test.

**Figure 7:**
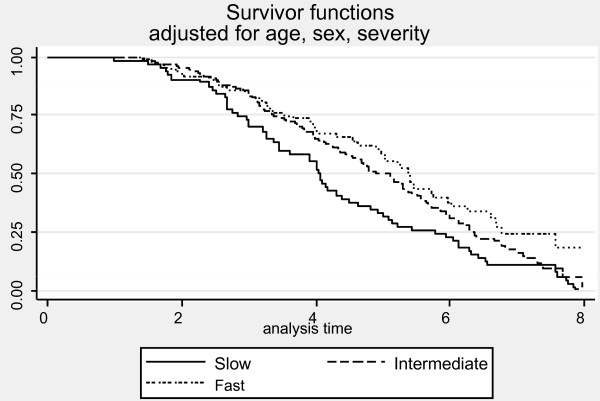
**Kaplan-Meier Survival curves by pre-progression group adjusted for age and sex**. HR for slow vs. fast = 0.62 (*P *= 0.024).

Average survival from first visit to death was 5.5 ± 2.7 years (median = 5.0 years). The median survival times for each of the pre-progression categories were: 4.7 years for slow, 4.1 years for intermediate, and 2.5 years for rapid progressors adjusted for age, sex, education and baseline severity (Figure 7). The results of Cox proportional hazards modeling indicated that slow progressors had significantly reduced mortality compared to fast progressors (HR = 0.62, 95% CI = 0.43 to 0.91, *P *= 0.024). Although intermediate progressors are distinguishable on the survival curves and the curves do not cross, the difference between the intermediate and fast progressors was not statistically significant (HR = 0.81 95% CI = 0.59 to 1.15, *P *= 0.24). Our study may have been underpowered to detect the small difference in survival between these two groups.

## Discussion

We have demonstrated in a large cohort of probable Alzheimer's disease patients that a simple, calculated, progression rate at the initial clinic visit is predictive of longitudinal performance on multiple cognitive and functional measures over time. These measures of cognition (ADAScog), attention and concentration (VSAT), global performance (CDR-SB), and activities of daily living (PSMS and IADL) are highly relevant to caregiving needs and to patient and caregiver quality of life, as well as representing measures commonly employed in clinical trials of AD treatments. The clearest and best maintained differences were observed between the slow progressors and those classified as fast progressors, who together constituted 54% of the population. On the ADAScog, for example, slow progressors maintained nearly a 10-point advantage over fast progressors (intermediate progressors maintained nearly a four-point advantage). Mixed effects regression modeling showed that, in effect, slow progressors are unlikely to *catch up *with fast progressors on standard outcome measures, even after up to seven years of observation. In fact, slow progressors diverge further from fast progressors over time on the ADAScog, while maintaining baseline differences on the VSAT, CDR-SB and IADL. Even though they did not differ in performance of basic ADL (PSMS) at baseline, slow progressors added disability in this area at a slower rate than fast progressors so that their performance diverged over time. Slow progressors also survived longer than fast progressors.

Intermediate progressors (46% of the patients) also maintained better cognition (ADAScog and VSAT) and function (IADL) compared to fast progressors, but they were less differentiated at baseline and sped up over time on a global measure, the CDR sum of the boxes score, and they were not differentiated at any time on the basic ADL (PSMS). The survival differences between intermediate and fast progressors were not significantly different, but our study may have been underpowered to detect a small difference. Our results suggest that prognostications based upon initial progression rate are most reliable for slow and fast progressors, but that long duration reliability of an intermediate progression rate may depend upon the patient's age and life expectancy at diagnosis. It would be safe to say that an intermediate progressor may remain so for several years, but that, if the patient lives for a long time after diagnosis, the rate may increase sufficiently to affect both abilities and survival.

Our methodology for classifying patients as slow, intermediate or rapid progressors could be easily employed by clinicians to calculate pre-progression rate at an initial clinic visit, using the MMSE score and a standardized approach to estimating duration [1,2]. The clinician could predict that a patient would generally progress slowly, moderately, or rapidly over several years. However, an important question remains as to whether these apparently intrinsic rates of disease progression can be modified, and this question must be resolved before the pre-progression approach is widely adopted for clinical purposes. In a separate paper, we demonstrated that persistent anti-dementia drug treatment impacts observed progression over time [20], an observation which is consistent with a recent analysis using a very different approach [21]. This effect of treatment persistence is significant in our mixed effects models which also include the pre-progression rate, indicating that treatment may provide benefit to patients regardless of their intrinsic progression rates. Treatment appears to alter slopes on measures which include the ones used in the current study, but we have not yet assessed whether the effect differs by pre-progression category.

Many investigators seek to validate biomarkers of disease progression, such as changes in hippocampal volume and serum and cerebrospinal fluid (CSF) biomarkers. The progression rates that are based upon clinical measures in such studies may need to be adjusted for early progression, or progression group, as well as for persistence of treatment, which could enhance observed correlations between valid biomarkers and clinical measures.

Our findings have important implications for the design and interpretation of AD clinical trials. Currently, parallel group studies count on randomization to yield comparable placebo and treatment groups. Pre-progression rates are not assessed -- yet imbalances across the treatment groups in this important variable could obscure true treatment differences, or could create apparent differences when there is no drug effect, especially in long duration clinical trials. Further, if our hypothesis that the persistency of anti-dementia drug treatment alters progression is correct, baseline differences in cumulative duration of drug use could create similar imbalances. Future clinical trials may benefit from gathering systematic data regarding individual symptom onset in order to perform a formal estimate of duration [2] and to calculate pre-progression rates [1], which could be used to stratify patients by progression group or as a covariate in the analysis. For those clinical trials that allow background treatment with marketed anti-dementia drugs while testing a new therapy against placebo, information about the quartile of persistence of anti-dementia treatment may also be needed to control for the impact of these variables in the analysis [20].

Our study has both strengths and limitations. It is a large study, including nearly 600 carefully diagnosed probable AD subjects followed for up to 15 years. Yet all of the subjects were followed at a single site, and we do not know how consistent our results would be in a multi-site study. Although we are located at a tertiary care center, we are one of the few clinics providing dementia care in the state, and we have few barriers to access, which together have led to an unusually diverse population [5]. Still we utilized a sample of convenience which may not be representative of the general AD population, and we do not know whether our results would be the same in a community based sample.

Further, because we did not randomize patients according to pre-progression rates at baseline, our inclusion of consecutive cases yielded groups of unequal size. We made appropriate adjustments to our analysis for clinical variables shown or hypothesized to influence rates of progression and survival in AD, including age, sex, education, premorbid IQ, hallucinations, delusions and extrapyramidal features. The progression group was an important predictor of longitudinal course even when these factors were taken into account.

Another strength of the study is our choice of standardized outcomes that are in clinical use and widely used in clinical trials. The importance of our findings is strengthened by the fact that the current data are internally consistent across multiple measures; progression groups maintained their differences on measures that included cognition, global performance, and activities of daily living. The fact that survival data were available for every subject and that survival time also differentiated the slow and fast progressors provides additional evidence for the clinical utility of the pre-progression rate.

## Conclusions

In conclusion there is a lack of data in the medical literature to guide clinicians and researchers in understanding the progression of Alzheimer's disease. Our data provide powerful evidence that prediction is possible, which addresses an important clinical need. Additionally, inclusion of the pre-progression rate in clinical trials for proposed AD therapies should enhance the power of such studies to find real treatment differences, and could reduce the duration of trials designed to assess disease-modifying therapies, which would also aid patients and those who care for them.

## Abbreviations

AD: Alzheimer's disease; ADAScog: Alzheimer's disease Assessment Scale cognitive subscale; ADMDC: Alzheimer's Disease and Memory Disorders Center; AMNART: American New Adult Reading Test; APO E: apolipoprotein E; CDR-SB: Clinical Dementia Rating Scale cognitive subscale; CSF: cerebrospinal fluid; DSM IV: Diagnostic and Statistical Manual of Mental Disorders; IADL: Instrumental Activities of Daily Living; MMSE: Mini-mental Status Examination; IQ: intelligence quotient; NIH: National Institutes of Health; NINCDS-ADRDA: National Institute of Nervous and Communicative Disorders and Stroke-Alzheimer's Disease and Related Disorders Association; PSMS: Progressive Self-Maintenance Scale; VSAT: Verbal Series Attention Task.

## Competing interests

The authors declare that they have no competing interests.

## Authors' contributions

RSD designed the study, drafted the manuscript, and obtained funding. RSD and SDR were involved in data acquisition and critical revision of the manuscript. RSD, VP, PM, and WC were involved in data analysis and critical revision of the manuscript. ED managed the database and was involved in data analysis and critical revision of the manuscript. All authors read and approved the final manuscript.
